# Identification of *Leishmania* species by high-resolution DNA dissociation in cases of American cutaneous leishmaniasis^☆^^☆☆^

**DOI:** 10.1016/j.abd.2020.02.003

**Published:** 2020-05-15

**Authors:** John Verrinder Veasey, Ricardo Andrade Zampieri, Rute Facchini Lellis, Thaís Helena Proença de Freitas, Lucile Maria Floeter Winter

**Affiliations:** a Dermatology Clinic, Faculdade de Ciências Médicas, Hospital da Santa Casa de Misericórdia de São Paulo, Santa Casa de Misericórdia de São Paulo, São Paulo, SP, Brazil; b Department of Physiology, Instituto de Biociências, Universidade de São Paulo, São Paulo, SP, Brazil; c Department of Pathological Anatomy, Hospital da Santa Casa de Misericórdia de São Paulo, São Paulo, SP, Brazil

**Keywords:** Diagnosis, Histology, *Leishmania braziliensis*, *Leishmania guyanensis*, *Leishmania infantum*, Leishmaniasis, Leishmaniasis, cutaneous, Leishmaniasis, mucocutaneous, Polymerase chain reaction

## Abstract

**Background:**

American cutaneous leishmaniasis is an infectious dermatosis caused by protozoa of the genus *Leishmania*, which comprises a broad spectrum of clinical manifestations depending on the parasite species involved in the infections and the immunogenetic response of the host. The use of techniques for amplification of the parasites DNA based on polymerase chain reaction polymerase chain reaction and the recent application of combined techniques, such as high-resolution DNA dissociation, have been described as a viable alternative for the detection and identification of *Leishmania* spp. in biological samples.

**Objectives:**

To identify the *Leishmania* species using the polymerase chain reaction high-resolution DNA dissociation technique in skin biopsies of hospital-treated patients, and compare with results obtained by other molecular identification techniques.

**Methods:**

A retrospective study assessing patients with suspected American cutaneous leishmaniasis seen at a hospital in São Paulo/Brazil was conducted. The paraffin blocks of 22 patients were analyzed by polymerase chain reaction high-resolution DNA dissociation to confirm the diagnosis and identify the species.

**Results:**

Of the 22 patients with suspected American cutaneous leishmaniasis, the parasite was identified in 14, comprising five cases (35.6%) of infection by *L. amazonensis*, four (28.5%) by *L. braziliensis*, two (14.4%) by *L. amazonensis* + *L. infantum chagasi*, two (14.4%) by *L. guyanensis*, and one (7.1%) by *Leishmania infantum chagasi*. In one of the samples, in which the presence of amastigotes was confirmed on histopathological examination, the polymerase chain reaction high-resolution DNA dissociation technique failed to detect the DNA of the parasite.

**Study limitations:**

The retrospective nature of the study and small number of patients.

**Conclusions:**

The method detected and identified *Leishmania* species in paraffin-embedded skin biopsies with a sensitivity of 96.4% and could be routinely used in the public health system.

## Introduction

Leishmaniasis is a chronic infectious-parasitic disease caused by several species of protozoa of the genus *Leishmania*. In Brazil, the species involved in infections with cutaneous manifestations are mainly *L*. *(V.) braziliensis* and *L*. *(L.) amazonensis*, followed by *L*. *(V.) guyanensis, L*. *(V.) lainsoni, L.* *(V.) shawi, L. (V.) naiffi* and *L.* *(V.) linderbergi*, with lesser incidence.^1^, ^2^, ^3^ More recently, isolated cases of American cutaneous leishmaniasis (ACL) caused by *Leishmania (L.) infantum chagasi* have been reported in patients with or without HIV co-infection in the central-western and southwestern regions of the country.^4^, ^5^

On the skin, the most common initial clinical presentation of ACL is a framed ulcer at the site of parasite inoculation. In this form, it is possible to find the parasite through direct search of the smear of the base of recent ulcers. However, there are other forms of cutaneous manifestations, such as verrucous, papular, nodular, sarcoid, and infiltrated and vegetative plaques, in which the presence of the parasite in the tissue is rare and histopathology is nonspecific, ranging from inflammatory lymphoplasmacytic infiltrate to granulomas with or without necrosis, thus making the differential diagnosis with other infectious, inflammatory, and even neoplastic diseases difficult.^6^, ^7^, ^8^, ^9^ Tissue immunohistochemistry with anti-*Leishmania* antibodies contributes to the diagnosis, but its sensitivity is around 66% of cases. A positive Montenegro skin test (>5 mm) can help, as it has a high predictive value. However, this test has some limitations: it is negative in the first months of infection and in anergic patients, and remains positive even after the disease is cured.^1^, ^6^

Therefore, leishmaniasis can be diagnosed through several methods, all of which present advantages and limitations. However, there is no gold standard diagnostic test. Microscopy is useful in endemic areas, but it has low sensitivity and depends on the assessment of an experienced pathologist; furthermore, it does not allow the identification of the parasite species. Serological tests can provide a quick diagnosis, are easy to interpret, and are relatively inexpensive. However, their sensitivity and specificity vary in different endemic regions; moreover, it is not possible to identify the species that caused the disease.^1^, ^3^, ^6^

Different methods with molecular approaches that explore specific characteristics of parasite DNA have been tested for the diagnosis of leishmaniasis. Initially, the analysis of the patterns of fragment generated by restriction enzymes, restriction fragments length polymorphisms (RFLPs), was the identification method for *Leishmania* and other trypanosomatids using DNA as a target.^10^, ^11^, ^12^ Another type of genotype analysis explored is the construction of probes used in molecular hybridization tests. This technique was used both in differentiation studies of strongly related species and for the diagnosis of infections.^13^, ^14^ The random amplified polymorphic DNA (RAPD) technique, which amplifies fragments with random primers through polymerase chain reaction (PCR), was also a technique adopted to analyze specific patterns capable of differentiating organisms of the *Leishmania* genus.^15^, ^16^ Due to numerous advantages, PCR-based tests are the main tools used in the detection and identification of *Leishmania*. This methodology allows the use of minute amounts of biological material, as well as the specific discrimination of organisms through the analysis of target polymorphisms. Different PCR-based techniques have been used in the identification of *Leishmania*, due to certain advantages, such as high speed and sensitivity/specificity, when compared with conventional techniques based on microscopy and cell culture.^17^

Several DNA sequences have been used as targets for the specific identification of *Leishmania*, such as kinetoplast DNA (kDNA),^18^ ribosomal DNA (rDNA),^13^, ^14^ and the g6pd gene, among other targets.^19^, ^20^ kDNA makes up about 15% of the parasite's DNA and is formed by a network of concatenated circular molecules (maxicircles and minicircles). Per organism, between 10,000 and 20,000 minicircles are found, ranging from 0.5 to 2.5 kb in size, and approximately 50 maxicircles from 20 to 35 kb, which encode mitochondrial proteins and ribosomal RNA of the organelle.^21^, ^22^ Among the targets described in the scientific literature, kDNA is one of the most used in molecular diagnosis. The high number of copies of the target per genome ensures a high sensitivity, representing the main advantage of the technique. However, the accuracy of the assays can be affected by the considerable heterogeneity of kDNA sequences.^23^

The characterization of ribosomal cistrons from different trypanosomatids allowed the creation of probes capable of identifying some organisms. At first. the existence of polymorphism in the spacer sequences of ribosomal genes in *Trypanosoma* spp. was demonstrated.^11^ The description of ribosomal cistrons from six species of *Leishmania* evidenced a characteristic pattern of these species, punctuated by the existence of a restriction site identified by the enzyme PvuII. This site, present in the coding sequence of the 18S rRNA or SSU, was explored in the construction of oligonucleotides capable of identifying the genus.^13^ Based on polymorphisms in the sequence of that same gene, probes that could be used to identify different groups of *Leishmania* have been described.^14^ Amplicon sequencing in this gene segment has been used to identify *Leishmania*. Two mutation points, found at positions 1714 and 1721 of the SSU rDNA sequence, are able to discriminate *(L.)* *amazonensis* and *L. (Viannia)* spp. Based on this strategy, several studies exploring the polymorphic characteristics of the SSU rDNA were carried out with different approaches, focusing on clinical, epidemiological, and ecological aspects, among others.^24^, ^25^, ^26^, ^27^, ^28^, ^29^, ^30^

Among the most used molecular identification techniques, the analysis by isoenzyme (zymodemes) electrophoresis is noteworthy. Glucose-6-phosphate dehydrogenase (G6PD) is one of the main enzymes used in the identification of *Leishmania* by zymodeme analysis. The polymorphism in the structure of these proteins is a reflection of polymorphisms at the gene level, which allowed the use of molecular markers capable of discriminating and quantifying *Leishmania* by conventional and real-time PCR.^19^, ^20^

In addition to these, other DNA targets described in the literature, such as the internal transcribed spacers (ITS) present in the rDNA cistron, the heat shock protein (Hsp70) gene, the cysteine proteinase (cpb) gene, among others, have been studied in Leishmania identification tests, based on the polymorphic characteristics of DNA.^17^

The simultaneous use of different targets has also been used in conventional PCR reactions, aiming to discriminate the pathogens of leishmaniasis. The combination of oligonucleotides designed from the sequence of the gene encoding the mini-exon added by trans-splicing to all trypanosomatid messenger RNAs (SL RNA) has been described as capable of discriminating, in a single PCR reaction, the species that cause the visceral form of the disease in one group and the species responsible for the cutaneous form of the disease in two other groups, which are distinct because they include species of the subgenera *L*. *(Leishmania)* or *L*. *(Viannia)*.^31^, ^32^

Recently, an important polymorphism was described in the hsp70 gene, associated with the analysis through high-resolution melting (HRM) analyses, a method capable of detecting differences in the nucleotide composition of specific PCR products that allows discriminating all species that circulate in Brazil and the most important species found in the Mediterranean, India, and North Africa.^33^ The application of this technique was shown to be useful in the diagnosis and identification of *Leishmania* in different samples from patients (fresh biopsies and in paraffin-embedded material) and from reservoir animals, such as dogs, and phlebotomine hosts.

Diagnostic tests that involve the detection of parasitic nucleic acids, mainly those based on specific DNA amplification such as PCR, are described as highly sensitive and specific options, with the potential to quantify and identify the infectious species.^8^, ^34^, ^35^, ^36^ HRM DNA analyses performed at the end of real-time PCR reactions are able to detect thermodynamic differences in the amplicon dissociation profile by determining specific signatures as a result of small differences in the nucleotide composition between the species. The PCR-HRM technique has been used to identify not only *Leishmania* species, but also other infectious agents, and has been described as a relatively cheap, fast, and robust tool for the detection and discrimination of *Leishmania* species in all endemic regions of the world.^34^, ^37^, ^38^

## Methods

This retrospective study was carried out at the dermatology clinic of a tertiary hospital in São Paulo, Brazil, analyzing the data of 22 patients diagnosed with ACL treated from January 2005 to December 2018. This study was approved by the ethics committee (CAAE: 48719115.0.0000.5479), and clinical, epidemiological, and complementary exam results were obtained from medical record analysis.

*Leishmania* spp. were identified through PCR-HRM, as previously described by this group,^33^ from a purified DNA sample from paraffin-embedded tissue obtained from the hospital's pathology laboratory. Samples were sent in a blind test criterion, even in cases where more than one sample was obtained from the same patient. Paraffin-embedded biopsies underwent a paraffin removal process with successive heated xylol baths, followed by ethanol baths, and subsequent hydration of the sample. The DNA from the biopsy samples was then extracted and purified using the DNeasy Blood & Tissue kit (Qiagen), according to the protocol suggested by the manufacturer. A first stage, called pre-amplification, was performed with primers based on the *Leishmania* hsp70 gene sequence that amplifies, by conventional PCR, a segment of DNA that contains polymorphic sites that can be analyzed by HRM. The pre-amplification products were then subjected to real-time PCR reaction, in which three distinct regions of the gene were amplified independently using the MeltDoc Master Mix kit (Life Technologies). HRM analyses were performed at the end of each real-time PCR reaction; the DNA dissociation profiles were generated by the High Resolution Melting software v. 3.0.1 (Life Technologies) and compared to profiles generated from DNA samples from reference strains of *Leishmania* spp. (L. (L.) *infantum chagasi* (MCER/BR/1981/M6445), *L. (L.) major* MHOM/IL/81/Friedlin), *L. (L.)* *amazonensis* (MHOM/BR/1973/M2269), *L. (L.) mexicana* (MNYC/BZ/62/M379), *L. (L.) lainsoni* (MHOM/BR/81/M6426), *L. (V.) braziliensis* (MHOM/BR/1975/M2903), *L. (V.)* *guyanensis* (MHOM/BR/1975/M4147), *L. (V.) naiffi* (MDAS/BR/1979/M5533), and *L. (V.)* *shawi* (MCEB/BR/84/M8408).

The results obtained by PCR-HRM were compared to methods already described in the literature, such as sequencing of a segment of the SSUrDNA gene, PCR-kDNA, and restriction fragment length polymorphisms (RFLPs) of the hsp70 gene.

## Results

**Figure 1:**
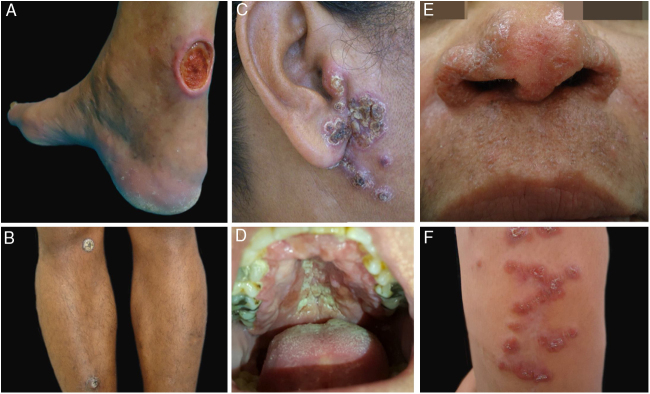
Clinical examples of patients with clinical lesions for which polymerase chain reaction high-resolution melting (PCR-HRM) assessment was able to determine the species of *Leishmania*. A, Patient 11 (*L. infantum chagasi*); B, Patient 13 (*L. amazonensis* + *L. infantum chagasi*); C, Patient 14 (*L. amazonensis* + *L. infantum chagasi*); D, Patient 7 (*L. brasiliensis*); E, Patient 3 (*L. amazonensis*); F, Patient 5 (*L. amazonensis*). (Figure not visible).

**Figure 2:**
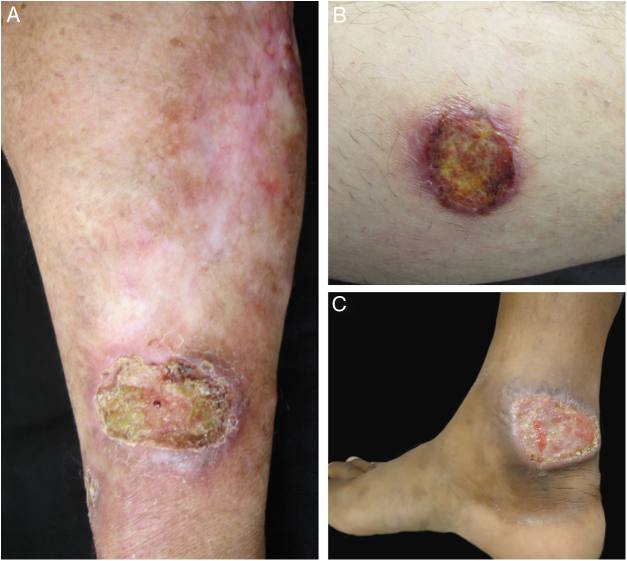
Clinical images of lesions for which polymerase chain reaction high-resolution melting (PCR-HRM) assessment was unable to determine the species of *Leishmania*. A, Patient 17, ulcer in the upper limb; B, Patient 21, ulcer in the lower limb whose biopsy showed the presence of amastigotes in the tissue; C, Patient 21, ulcer in the lower limb.

During the study period, 22 patients with suspected diagnosis of ACL were treated, of whom 12 were male and 10 were female. Age ranged from 13 to 78 years, with a mean of 45.72 ± 18.41 years and a median of 43.5 years. The duration of the skin lesion ranged from one to 108 months, with a mean of 28.31 ± 35.49 months and a median of ten months. The most common clinical presentation was skin ulcers (13 patients), followed by mucosal ulcers (four patients with nasal lesions and two with oral lesions), papular skin lesions (two patients), and one nodular skin lesion in one patient (Figure 1, Figure 2).

The epidemiological, clinical, and histopathological characteristics, as well as the results of the direct search of the parasite in the injured tissue of the 22 patients included in the study, are specified in table 1.

**Table 1:** Clinical, epidemiological, and laboratory characteristics of the cases studied.

Sample characteristics		Lesion characteristics			Evidence of agent		Complementary tests				
Patient	Origin (state)	Clinical presentation	Tissue/location	Time-length of lesion (months)	Direct exam	Histopathology	PCR SSU	PCR	SSU	hsp70	hsp70
						IHQ		kDNA	Seq.	RFLP	HRM
1	MG	Ulcer	Skin/limb	4	+	NP	+	+	NP	*L. amazonensis*	*L. amazonensis*
2	PR	Ulcer	Skin/limb	4	+	+	+	+	NP	*L. amazonensis*	NP
3	BA	Ulcer	Nasal mucosa	72	−	NP	+	+	NP	*L. amazonensis*	*L. amazonensis*
4	BA	Papule	Skin/Nose	24	+	−	+	+	*L. amazonensis*	*L. amazonensis*	NP
5	SE	Nodules	Skin/limb	108	−	NP	+	+	*L. amazonensis*	*L. amazonensis*	*L. amazonensis*
6	BA	Ulcer	Skin/Ear	6	+	NP	+	+	NP	NP	*L. braziliensis*
7	MG	Ulcer	Oral mucosa	3	NP	+	+	+	*L. (Viannia)*	NP	*L. braziliensis*
8	MG	Papule	Skin/Periocular	18	+	+	+	+	NP	NP	*L. braziliensis*
9	BA	Ulcer	Skin/limb	12	+	NP	+	NP	NP	NP	*L. braziliensis*
10	BA	Ulcerated plaque	Skin/limb	12	NP	−	+	−	NP	NP	*L. guyanensis*
11	AL	Ulcer	Skin/limb	96	−	NP	+	−	NP	NP	*L. guyanensis*
12	BA	Ulcer	Skin/limb	2	+	NP	+	+	*L. i. chagasi*	*L. i. chagasi*	*L. i. chagasi*
13^a^	RN	Ulcerated plaque	Skin/limb	7	NP	+	+	+	*L. amazonensis/L .i. chagasi*	*L. amazonensis/L .i. chagasi*	*L. amazonensis/L. i. chagasi*
14&	BA	Ulcer	Skin/Ear	5	+	NP	+	+	*L. amazonensis/L .i. chagasi*	*L. amazonensis/L .i. chagasi*	*L. amazonensis/L. i. chagasi*
15	SP	Ulcer	Skin/limb	5	NP	−	−	NP	NP	NP	NP
16	BA	Ulcer	Oral mucosa	12	−	NP	−	NP	NP	NP	NP
17	BA	Ulcer	Skin/limb	8	−	NP	−	NP	NP	NP	NP
18	BA	Ulcer	Nasal mucosa	60	−	NP	−	NP	NP	NP	NP
19	MG	Ulcer	Skin/limb	4	−	NP	−	NP	NP	NP	NP
20	BA	Ulcer	Nasal mucosa	60	−	NP	−	NP	NP	NP	NP
21	SP	Ulcer	Skin/limb	5	NP	+	−	NP	NP	NP	NP
22	SP	Ulcer	Nasal mucosa	96	−	NP	−	NP	NP	NP	NP

NP, not performed, and molecular tests applied to two different samples, one indicating the presence of *L. amazonensis* and the other of *L. infantum chagasi*. RFLM, restriction fragment length polymorphisms; PCR, polymerase chain reaction; HRM, high-resolution melting.

a Molecular tests indicated the presence of the two parasites in the same sample.

For diagnosis, smears were performed on the lesions of 17 patients (eight positive) and skin biopsies in all 22 patients (14 positive); all samples were assessed by routine staining (hematoxylin–eosin), and an immunohistochemical study for detection of *Leishmania* was conducted in eight patients. In ten patients, more than one biopsy was performed in order to diagnose the disease. In all, 38 blocks were analyzed by histology, PCR-SSU, PCR-kDNA, SSU-Seq, PCR hsp70-RFLP, and PCR hsp70-HRM (Table 1).

Eleven patients presented amastigotes on smear and/or histological examination, and only two patients were positive in both tests. In 11 patients, no amastigotes forms were observed in either test (Table 1).

Of 22 patients with suspected ACL treated in the period, 14 had the parasite species identified by SSU-Seq, PCR hsp70-RFLP, or PCR hsp70-HRM, with five cases (35.6%) of infections by *L. amazonensis*, four (28.5%) by *L. braziliensis*, two (14.4%) by *L. amazonensis* + *L. infantum chagasi*, two (14.4%) by *L. guyanensis*, and one (7.1%) by *Leishmania infantum chagasi*. It is interesting to note that the sequencing of the SSU fragment does not allow the identification of species of the sub-genus *L.* *(Viannia)*, but there was a coincidence of the identified species, either within this sub-genus, or in cases where there was a double identification of *L. amazonensis* + *L. infantum chagasi* (Table 1).

From the total of samples from 11 patients in which amastigotes were detected through direct research on the smear of the lesion and/or histopathological examination of a skin fragment, it was possible to identify the species of the parasite in ten of them, and only one was negative by the molecular methods employed. In contrast, of the 11 negative samples by smear and/or histopathological exams, the protozoan was identified in four (Table 2). It is noteworthy that one of these four patients (patient 5) presented nodular skin lesions of difficult clinical and pathological diagnosis for 108 months (Fig. 1 and Table 1).

**Table 2:** Results of evidence of the parasite by direct examination on lesion smear and/or histopathological examination and identification of *Leishmania* spp. through PCR-HRM, correlated with the clinical presentation.

Muco-cutaneous lesion		Parasite presence (DE/HE)		Absence of parasite (DE/HE)		Total
		Positive PCR-HRM	Negative PCR-HRM	Positive PCR-HRM	Negative PCR-HRM	
Skin	Skin ulcer	7	1	2	3	13
	Papules	2	0	0	0	2
	Nodules	0	0	1	0	1
Mucosa	Oral ulcer	1	0	0	1	2
	Nasal ulcer	0	0	1	3	4
Total		10	1	4	7	22

DE, direct examination; HE, histopathological examination; PCR, polymerase chain reaction; HRM, high-resolution melting.

PCR-SSU was positive for 14 of the patients, and the species were identified in all of them, either by SSU-Seq, PCR hsp70-RFLP, or PCR hsp70-HRM. Moreover, PCR-kDNA was performed for these 14 patients, resulting in 12 positive samples.

## Discussion

Even today, it is still difficult to make an accurate diagnosis in patients with suspected cutaneous and/or mucous leishmaniasis when no parasites are found in the lesions. Several complementary exams are requested to attempt to reach a diagnosis and exclude other diseases such as mycobacteriosis, sarcoidosis, lymphomas, and deep mycoses, among others.^1^, ^6^, ^8^ Thus, it is important to have a quick diagnostic test, with high specificity and sensitivity, to avoid excessive tests and consultations, as well as the need for therapeutic tests with undesirable side effects. In recent years, the use of PCR to determine the parasite has evolved considerably; the use of this technique together with HRM, targeting the *Leishmania* hsp70 gene, allows the laboratory identification of all species with high specificity and sensitivity.^34^, ^37^, ^38^

In addition, the present results highlight the fact that, even in the case of a public tertiary hospital of reference in the largest city in Brazil, access to simple diagnostic tests is not always available. Of the 22 cases studied, for only eight cases (36.3%) was it possible to perform an immunohistochemical study for *Leishmania*. A direct search for the parasite in lesion smear stained with Giemsa was the most accessible test, performed in 17 patients (77.2%).

The presence of the parasite is demonstrated by detecting its DNA in potentially sensitive tests, since positivity is the result of exponential target amplification by PCR. In fact, the PCR tests applied to 14 of the 22 patients were positive in the SSU assessment (63.64%) and 12 were positive in the kDNA assessment (54.55%), and these were also positive for PCR-SSU. However, these targets do not allow discrimination of the parasite species.

There was agreement in the identification of the species through SSU-Seq, hsp70-RFLP, and hsp70-HRM; however, the first two are laborious tests, which require the manipulation of the PCR product, in addition to a trained technician to perform and analyze the results.

In a patient in whom the presence of amastigotes was detected by histopathology, the PCR-SSU and hsp70-HRM tests were negative. A possible failure in detecting the parasite's DNA can have biological or physicochemical explanations. The first is based on the fact that cutaneous and mucocutaneous lesions caused mainly by *L. braziliensis* have as a common characteristic the scarcity or absence of parasites, hindering laboratory diagnosis. The second is based on the fact that biological samples that undergo fixation for histological analysis are subject to DNA degradation or polymerase enzyme inhibition, mainly due to exposure to formalin or reagents used in sample preparation.^1^, ^3^, ^6^, ^38^ In the case of the patient with evidence of amastigotes by immunohistochemistry, the sample was subjected to a host DNA amplification test by conventional PCR, and the target was amplified. This test is routinely performed to assess the quality of the DNA and the interference of the possible presence of polymerase inhibitors in the samples. This result suggests, then, that the absence or extreme scarcity of parasites in this sample can be a convincing explanation for the negativity of the parasite detection and identification test.

The identification of *L. amazonensis* in lesions of five patients from Bahia (two), Sergipe (one), Paraná (one), and Minas Gerais (one) initially surprised the authors, as in these states *L. braziliensis* is the species most commonly related to cases of muco-cutaneous leishmaniasis. *L. amazonensis* is classically considered restricted to Amazonian areas; it is transmitted through wild vectors and rodents are the main reservoirs. However, changes in the epidemiological profile of this species have recently been described. In the state of Bahia, where the presence of *L. braziliensis* is more common, the presence of *L. amazonensis* causing skin lesions, and more rarely, mucosal lesions, was demonstrated in 1988 through panels of monoclonal antibodies.^39^ In other regions such as Mato Grosso do Sul, Santa Catarina, Paraná, and Rio de Janeiro, isolated human cases of cutaneous leishmaniasis by *L. amazonensis* have been identified.^40^ This species has also been identified in dogs in the state of São Paulo^41^, ^42^ and more recently in endemic areas in southeastern Brazil^43^, ^44^ Thus, the authors believe that an unknown proportion of cases of cutaneous leishmaniasis outside the Amazon may be caused by *L. amazonensis,* and that further epidemiological studies are needed since evidence indicates that the transmission profile of cutaneous leishmaniasis may be changing.

The finding of a mucosal lesion caused by *L. amazonensis* was also unexpected. Rarely have human cases been described in the literature, despite having been reproduced experimentally in rats.^45^ The identification of *L. guyanensis* in two patients from Northeastern Brazil was another unexpected finding, since this species has a transmission cycle that is apparently limited to the Northern region.^46^, ^47^ However, it is noteworthy that the geographical origin mentioned in the medical records does not indicate where the patients were infected, since they may have moved across the different regions of the country, and more accurate information is often scarce in retrospective studies. Conversely, the occurrence of these species outside endemic areas warns to the possibility of a new epidemiology of the disease. It is unknown whether their geographic distribution has expanded or whether these species have been distributed in the national territory for some time, and environmental changes are increasing the exposure of animals (such as dogs) and humans to parasites in areas where they had not been described. New methods for identifying the species of parasites allow their geographical distribution to be reviewed, contributing to the understanding of the epidemiology of the disease.

The identification of a case with *L. infantum chagasi* is considered a rarity in ACL in Brazil, since this species is generally related to visceral presentations.^4^, ^5^ Isolated cases have been described in association with HIV infection, in young patients, and even one in an urban area of Rio de Janeiro, without any associated comorbidity,^5^ as was one of the present cases. Albeit rare, mixed infections caused by more than one species of *Leishmania*, or even trypanosomatids of other genera parasitizing the same host, have been described in the literature.^48^ In 2002, Martinez et al. described the first case of detection of *L. amazonensis* and *L. infantum chagasi* in the same lesion in a Bolivian patient, a situation similar to that identified in one of the present cases.^49^ These two species were found in another patient, but in different samples, which does not allow inferring whether they were two different temporal infections or a double initial infection.

This study has limitations due to its retrospective nature and small sample size; however, by expanding the observations to more cases, it may point to new paradigms in the identification of *Leishmania* species related to the different geographical areas and the forms of the disease. DNA samples from patients with a negative final diagnosis for leishmaniasis should also be considered informative. There was an agreement between the negative results and the absence of amastigotes in 19 paraffin-embedded tissue blocks from eight patients. In practical terms, a reliable negative result can prevent the collection of more samples, due to failure of diagnosis, in addition to preventing the patient from undergoing unnecessary chemotherapy.

Ideally, PCR exams for the detection and identification of *Leishmania* should be accessible to most health care services in Brazil. Prior to this study and access to species identification by hsp70-HRM in paraffin-embedded tissue blocks, patients were treated at this service based on clinical (presence of mucocutaneous lesions) and epidemiological data, as well as complementary exams, when available. In the present cases, it was demonstrated that access to complementary exams is not routine, and that in those without amastigotes, diagnosis confirmation is difficult; thus, therapeutic testing with possible side effects is usually indicated, which often causes discomfort and suffering to the patient.

The identification of *Leishmania* species is important for the choice of treatment.^1^, ^3^, ^47^ *L. braziliensis* can lead to mucosal involvement, even in patients who had a single ulcer,^3^, ^6^ and there is a possibility of visceral involvement in patients with skin lesions caused by *L. infantum chagasi*.^4^, ^5^, ^50^ Thus, in these cases, systemic treatment should be preferred over topic treatments such as intralesional infiltrations with meglumine antimoniate, recommended for the treatment of single ulcerated lesions on the skin.^3^

## Conclusions

The diagnosis of leishmaniasis is one the greatest challenges in the fight against the disease. The correct identification of the *Leishmania* species in paraffinized samples of skin and mucosa is an important source of data for ecological and epidemiological studies, as well as for studies of the biology of the parasite and its interaction with the host, and may be indicative of the design of the therapeutic protocol.

Of the three tests used to identify the species, two are very laborious and require a trained technician to interpret results. The fact that the hsp70-HRM result is provided by a machine not only allows for automation, but also eliminates potential contamination resulting from the manipulations necessary for the analysis of results obtained by conventional PCR, as this technique is based on analysis of the PCR product in sealed tubes, which do not require handling.

This study was able to identify the parasite species in clinical samples in 10/11 patients with positive amastigotes. Furthermore, it demonstrated the presence of *Leishmania* in 4/11 patients in whom amastigote forms had not been found in the studied samples and definitely ruled out the diagnosis of leishmaniasis in 7/11 patients with no parasites on direct exams.

In addition to *L. braziliensis*, *L. guyanensis*, *L. amazonensis,* and *L. infantum chagasi*, an association of the latter two species was detected. In practice, these results indicate that the occurrence of rare cases may be more common than previously described.

The authors believe that prospective studies should be conducted with larger populations; the use of the hsp70-HRM technique with material obtained from smears is also suggested, as it would allow studies in remote regions of the country.

## Financial support

None declared.

## Authors’ contributions

John Verrinder Veasey: Statistical analysis; approval of the final version of the manuscript; conception and planning of the study; elaboration and writing of the manuscript; obtaining, analyzing, and interpreting the data; effective participation in research orientation; intellectual participation in propaedeutic and/or therapeutic conduct of studied cases; critical review of the literature; critical review of the manuscript.

Ricardo Andrade Zampieri: Approval of the final version of the manuscript; conception and planning of the study; elaboration and writing of the manuscript; obtaining, analyzing, and interpreting the data; effective participation in research orientation; critical review of the literature; critical review of the manuscript.

Rute Facchini Lellis: Obtaining, analyzing, and interpreting the data; intellectual participation in propaedeutic and/or therapeutic conduct of studied cases.

Thaís Helena Proença de Freitas: Statistical analysis; approval of the final version of the manuscript; conception and planning of the study; elaboration and writing of the manuscript; obtaining, analyzing, and interpreting the data; effective participation in research orientation; intellectual participation in propaedeutic and/or therapeutic conduct of studied cases; critical review of the literature; critical review of the manuscript.

Lucile Maria Floeter Winter: Statistical analysis; approval of the final version of the manuscript; conception and planning of the study; elaboration and writing of the manuscript; obtaining, analyzing, and interpreting the data; effective participation in research orientation; critical review of the literature; critical review of the manuscript.

## Conflicts of interest

None declared.
